# Janus Kinase-2 V617F Mutation and Antiphospholipid Syndrome in Cerebral Sinus Venous Thrombosis: Natural History and Retrospective Bicenter Analysis

**DOI:** 10.3389/fneur.2022.783795

**Published:** 2022-04-14

**Authors:** David Orion, Ze'ev Itsekson-Hayosh, Shlomi Peretz, Rom Mendel, Gal Yaniv, Moshe Attia, Drorit Grizim-Merkel

**Affiliations:** ^1^Department of Stroke and Neurovascular Disorders, Chaim Sheba Medical Center, Ramat Gan, Israel; ^2^Department of Neurology, Sackler Faculty of Medicine, Tel Aviv University, Tel Aviv, Israel; ^3^Department of Neurology, Rabin Medical Center, Petah Tiqwa, Israel; ^4^Department of Radiology, Interventional Neuroradiology and Radiology Units, Sheba Medical Center, Sackler Faculty of Medicine, Tel Aviv University, Tel Aviv, Israel; ^5^Department of Neurosurgery, Sheba Medical Center, Affiliated to Sackler Faculty of Medicine, Tel Aviv University, Tel Aviv, Israel; ^6^Hematology Institute, Sheba Medical Center, Sackler Faculty of Medicine, Tel Aviv University, Tel Aviv, Israel

**Keywords:** sinus venous thrombosis, JAK-2 mutation, thrombophilia, stroke workflow, APLA

## Abstract

**Background:**

Cerebral sinus venous thrombosis (CSVT) is a rare neurovascular entity, usually associated with acquired or genetic hypercoagulable states. In up to 30% of the cases it remains idiopathic. Bone marrow proliferation disorders that are associated with Janus Kinase 2 V617F mutation (JAK-2) are known causes of the systemic and cerebral thrombosis—at times despite normal blood counts—for which hematologic treatment exists. However, JAK-2 prevalence in the CSVT cases is not clear.

**Methods:**

In this retrospective analysis, data of 236 patients with CSVT admitted to two tertiary centers between 2010 and 2020 were analyzed, with emphasis on laboratory and imaging data and clinical and interventional outcomes.

**Results:**

A total of 236 patients were included in the analysis. The patients' median age was 42 years and the average age was 44 years (±19 years), with 59% female patients. JAK-2 positivity rate was 18% (among 77 patients tested for the mutation). Patients with normal blood counts on presentation comprised 36% of the JAK-2 positive cases. Other hypercoagulability states were also investigated, with the antiphospholipid syndrome (APLA) showing the highest prevalence (11%) followed by other etiologies including oral contraceptive use, Factor V Leiden, prothrombin mutation, and malignancy. Selected JAK-2, APLA, and prothrombin mutation cases showed a more severe clinical course.

**Conclusion:**

JAK-2 mutation is underdiagnosed and its screening may be warranted in the cases of idiopathic CSVT, even despite normal blood counts, to allow disease-modifying treatment and blood cell count monitoring. JAK-2, APLA, and prothrombin mutation may be associated with a more complicated clinical course.

## Introduction

Cerebral sinus venous thrombosis (CSVT) is a rare neurovascular entity that can lead to devastating outcomes (1–3). This condition commonly affects young patients and has various etiologies, including local compression with subsequent venous occlusions such as tumors or trauma, infection or pharmacological therapy, and genetic or acquired procoagulant states (4). However, in up to one-third of cases, the etiology remains cryptogenic, requiring 6–12 months to lifelong anticoagulant therapy, depending upon the clinical presentation, demographics, and episodes of recurrence (5).

Polycythemia vera or essential thrombocytemia (PV and ET) are a JAK-2 V617F, acquired somatic mutation disorders (JAK-2) that is associated with bone marrow function disorders (6). These conditions are associated with increased blood viscosity and arterial or venous thromboembolism (7–9). The hallmark of these conditions is abnormalities in routine blood cell count (hematocrit, red blood cell count or hemoglobin levels, and platelet count). Following positive molecular testing for known mutations (JAK-2, CALR2, and MPL), at times followed by a bone marrow biopsy to rule out hematologic malignancy, a treatment protocol can be implemented in order to control the dysfunction of the bone marrow (10–12). In the cases of PV- or ET-related thrombosis, antiplatelet treatment is usually sufficient, combined with specific hematological treatment such as hydroxyurea or JAK-2-specific inhibitors (13). Therefore, control of blood counts and subsequently of blood viscosity, especially in cases of PV, can significantly lower the risk of recurrent thrombotic events and monitor malignant transformation. Using the current molecular diagnosis techniques of PV and ET is easy and accessible. However, the workup for these conditions is warranted today mainly in cases of abnormal blood counts, either as an incidental finding or on presentation as an acute thrombotic event (14, 15). The implication of this common practice is that in most cases of idiopathic thrombosis in patients with normal blood count JAK-2 workup is not performed routinely. This exposes these patients to potential misdiagnosis and deprives them of the disease-controlling treatment, possibly leading to recurrent thrombotic events.

In this study, we present a retrospective analysis of patients with CSVT admitted to the stroke unit in two tertiary centers during a period of 10 years. We show that despite normal blood counts, a proportion of idiopathic CSVT patients were diagnosed with either ET or PV. We also discuss pathophysiological mechanisms, natural history, and outcomes of these and other unprovoked patients with CSVT in this cohort.

## Materials and Methods

### Patients

The Chaim Sheba Medical Center (CSMC) and the Rabin Medical Center (RMC) medical files were retrospectively queried for adult patients above the age of 18 who were diagnosed with cerebral sinus thrombosis between the years 2010 and 2020. Neuroimaging was reviewed by a stroke neurologist to confirm cerebral venous thrombosis based on CTA or MRA. In cases in which source images were not available for review, we relied on formal neuroradiology reports confirming CSVT; however, exact venous involvement could not be determined directly and hence was not included in the analysis. The retrospective evaluation of the patients' files was approved by the Ethical Committees of CSMC and RMC (Helsinki Committee Approval Numbers 6067-19 and 0452-18, respectively). Clinical, laboratory, and imaging data were gathered and documented by an experienced stroke neurologist in each center, including the incidence of venous ischemia, bleeding, papilledema, need of urgent venous thrombectomy, and follow-up imaging. Severe outcomes according to available data were defined as intracranial hemorrhage, need of surgical intervention (thrombectomy of craniotomy), venous infarction, epileptic disorder, recurrent hospitalizations, and recurrent thrombosis events.

### Imaging

All our patients underwent brain CT, MRA, or both.

In most cases, MRI, including MRA, was performed during the first few days of presentation. Some of the patients underwent a follow-up scan.

CT imaging was performed either on a General Electric Revolution 256 scanner or a Phillips iCT 256 station. Injected contrast media was based on Iohexol solution (Omnipaque 350 mg I/ml) by General Electric Healthcare.

Magnetic resonance imaging was acquired on a Philips Ingenia 3.0 Tesla scanner; injected contrast media included gadoteric acid solution (Dotarem at a concentration of 9.1 g/100 ml).

### Laboratory Studies

All the patients underwent basic and advanced laboratory studies based on routinely accepted studies at the CSMC and RMC Laboratory Divisions, including rheumatologic and hypercoagulable conditions (antiphospholipid antibody screening, circulating anticoagulant panels, protein C and S levels, activated protein C ratio, Factor V Leiden and Prothrombin mutation genetic panels, complement levels, anti-RO, anti-LA, anti-Sm and anti-RNP levels, ANCA and ANA panels, and ESR and CRP levels). Specifically, the serologic positivity criteria were based on the institutions' accepted threshold values for the antibody titers, including APLA titers and prolonged coagulation times for lupus anticoagulant tests. These were approved by a specialist in rheumatology and immunology as part of the accepted, standard clinical care in the participating institutions.

Selected patients also underwent a lumbar puncture to measure intracranial pressure and assess the cerebrospinal fluid (CSF) profile for inflammatory conditions and specifically vasculitis. Selection of patients for molecular screening for JAK-2 mutation was limited to the approved indications at the time of data collection in the involved institutions, based either on abnormal blood counts on presentation or in the cases of extensive thrombosis (either bilateral or involving more than two major venous sinuses) and fulminant course with otherwise negative workup.

### JAK-2 Mutational Analysis

Genomic DNA was isolated with the G-DEX™IIc Genomic DNA Extraction Kit (iNtRON Biotechnology). JAK-2 mutation assay for V617F was assessed using a qualitative, real-time, PCR-based allelic discrimination assay, as described previously (16, 17). PCR was performed on QuantStudio 5 Real-Time PCR analyzer (Applied Biosystems) in two separate tubes for normal and mutated alleles. In total, fifty nanograms of genomic DNA were amplified in a 40-cycle PCR at an annealing temperature of 62°C. All the reactions were performed in a final volume of 20 ul contacting SYBER Absolute ROX mix (Thermo Scientific©). PCR results were analyzed applying the delta Ct method. A cutoff of 1% or more of mutate alleles was chosen to define an individual as positive. The nominal estimated cost per test is estimated at 100 USD, which was fully financed by the public healthcare system.

### Statistical Analysis

Qualitative and quantitative analyses were performed on the data. Analyses were performed on the clinical, laboratory, and imaging variables. Relative risk values in APLA and the JAK-2 subgroups were calculated using the Altman Method test and compared with the prevalence of specific outcomes and clinical variables in the general cohort. Multivariate analysis was used to neutralize specific demographical/clinical biases in the analysis of the etiological subgroups. Cutoff for statistical significance was set at *p* < 0.05. The analyses were performed using Excel Statistical functions (Microsoft Corporation), Graph-Pad Prism software (San Diego, California, USA), and MedCalc online freeware (MedCalc Ltd.).

## Results

Between the years 2010 and 2020, 236 patients were admitted to two tertiary centers in central Israel with a radiologically confirmed diagnosis of sinus venous thrombosis. Clinical, radiological, and demographical data are shown in Table 1. The patients' median age was 42 years, with 59% female patients. In 91 patients (38.6%), the etiology of thrombosis remained idiopathic despite extensive workup as detailed above. Table 2 summarizes the etiological data of the cohort. In 79 cases (33.3%), either acquired or genetic hypercoagulable state was identified. Twenty-three cases (9.7%) were associated with antiphospholipid syndrome and only 5 cases were associated with a systemic inflammatory disease such as Behcet's disease or systemic lupus erythematosus. Twenty-nine cases (9.3%) were attributed to a known or a newly diagnosed malignancy, either hematologic, systemic, or a primary intracranial tumor. Fourteen cases (5.9%) were attributed to an infection (systemic, local, or CNS), and another 14 cases (5.9%) were attributed to trauma or iatrogenic causes. Thirty-seven patients (15.7%) were active smokers and 46 (19.5%, corresponding to 32.8% of the female population in the cohort) were on concurrent oral contraceptive treatment.

**Table 1:** Clinical, demographical, and laboratory data of patients with CSVT (%**—**calculated of total, 236 patients' cohort).

**Demographics and clinical data**	**N**	**%**
Mean age = 44 ± 19 y		
Median age = 42 y		
Female	140	59.3
Smoking	37	15.7
Underlying chronic conditions	27	11.4
Oral contraceptives	46	19.5
Symptoms and signs		
Headache	109	46.2
Visual disturbances	12	5.1
Focal signs	33	14
Seizure	21	8.9
Decreased level of consciousness	12	5.1
Papilledema	54	22.9
Other	7	3
Incidental finding	13	5.5
Thrombus location (by direct review)		
Superior sagittal sinus	24	10.2
Transverse sinus	11	4.7
Sigmoid sinus	9	3.8
Jugular vein	4	1.7
Cavernous sinus	1	0.4
Extensive thrombosis	148	62.7
Deep and cortical veins	11	4.7
Radiological report only	28	17.8
Treatment		
Warfarin or LMWH	186	78.8
DOAC's	5	2.1
Antiplatelets	1	0.4
Missing data	29	12.3
None	15	6.4
Imaging outcome (6–12 months follow-up)		
No recanalization	12	5.1
Partial recanalization	61	25.8
Full recanalization	91	38.6
Missing data	72	30.5

*CTA, computed tomography angiography; MRA, magnetic resonance angiography; LMWH, low-molecular-weight heparin; DOAC's, direct oral anticoagulants*.

**Table 2:** Etiological data of patients with CSVT, with basic demographic and outcome data.

**Etiologies**	**N**	**% Of cohort**	**Average age**	**% Females**	**%Partial recanalization**	**% Intracranial hemorrhage**	**% Focal deficits or altered level of consciousness on presentation**	**% Re-hospitalization**
None identified	91	38.6						
Oral contraceptives	46	19.5	29	100	15.2		8.6	15.2
Pregnancy and Puerperium	10	4.2	28.6	100	20		0	10
Thrombophilia								
APLA	23	9.7	41	78.2	52.4	8.7	21.8	28.6
JAK-2	14	5.9	36	85.7	35.8	38.5	21.4	28.6
FVL	10	4.2	39.1	50	30	10	10	20
MTHFR mutation	2	0.8	na	na	na	0	0	na
Prothrombin mutation	9	3.8	43.9	44.4	33.3	**33.3**	44.4^*^	44
Protein S or C deficiency	9	3.8	40.4	88.9	11.1	**11.1**	44.4^*^	22.2
Other/combined	12	5.1	38.3	58.3	33.3	33.3	0	41.7
Malignancy								
Hematological	2	0.8	na	na	na	50	50	na
Systemic	10	4.2	65.4	50	50	20	10	30
CNS	10	4.2	51.2	50	30	0	20	20
Infection	14	5.9	60.3	42.3	7.1		21.4	0
CNS	6	2.5				0		
Local	5	2.1				0		
Systemic	3	1.3				0		
Systemic inflammatory disorders	5	2.1	na	na	na	0	na	na
Dehydration	1	0.4	na	na	na	0	na	na
Trauma	13	5.5	46.1	38.5		0	7.7	
Other	11	4.7	na	na	na	0	na	na

* *High prevalence of adverse outcomes in the prothrombin mutation and Protein S/C deficiency subgroup **(Relative risk = 2.46 confidence interval 1.13–5.37, p = 0.023)***.

*na, not applicable due to small sample size; APLA, Antiphospholipid Antibody Syndrome; FVL, Factor V Leiden; JAK2, Janus Kinase 2; MTHFR, methylenetetrahydrofolate reductase; CNS, central nervous system. Bold values refers to statistically significant values*.

In respect to the radiological outcomes, out of 198 (83.9%) patients that underwent a follow-up CTA or MRA, 91 (38.6%) patients achieved full recanalization of the thrombosed sinuses, while in 12 (5.1%) patients no recanalization was achieved.

Regarding the clinical course, the most common presenting sign was headache (109 patients, 46.2%). Sixty-six (27.8%) patients presented with either focal signs, seizures, or decreased consciousness. Visual changes were reported only in 12 patients (5%). Papilledema was found in 54 out of 120 patients who underwent fundoscopic examination (45 and 22.9% of the total cohort). In 57.2% of patients, the general clinical course was benign and did not result in neurological sequelae. In regard to the severe outcome as defined in the methods section, the post-thrombosis epileptic disorder was documented in 29 patients (12.3%). Twenty-three (9.7%) patients required surgical intervention, most commonly VP shunting due to chronic papilledema, followed by optic nerve sheath fenestration. Finally, only 3 cases required emergent venous thrombectomy. In 42 (17.8%) patients, the thrombosis resulted in venous parenchymal hemorrhage. Forty-nine (20.7%) patients required repeated hospitalizations. Additionally, in our cohort, only 1 true venous infarction case was documented (in patient with APLA), and only 2 cases of confirmed recurrent thrombosis were seen (1 in the JAK-2 positive patient and 1 associated with idiopathic thrombosis). Prothrombin mutation, JAK-2 mutation, and APLA syndrome were among the leading procoagulable states, also associated with a trend toward more severe outcomes (as presented in Tables 3, 4).

**Table 3:** Subgroup analysis of JAK-2-positive patients.

**Demographics and clinical data JAK-2 positive patients (*n* = 14)**	**N**	**%**	**RR (CI, *p* value) compared to general cohort**	**RR (CI, *p* value) compared to JAK-2 tested *negative* patients**
Mean age = 36 ± 18 y				
Median age = 29 y			ns	ns
Female^*^	12	85.7	2.34 (1.81–3.02, **<0.001**)	1.86 (1.32–2.62, **0.0004**)
Smoking	3	21.4	ns	ns
Underlying chronic conditions	2^#^	14.3	1.4 (0.37–5.37, 0.6)	na
Oral contraceptives	2	14.8	0.86 (0.23–3.2, 0.8)	na
Symptoms and signs				
Headache	9	64.3	1.97 (1.37–2.85, **0.0003**)	2.02 (1.19–3.45, **0.009**)
Visual disturbances	0	0	na	
Focal signs	3	21.4	1.05 (0.35–3.2, 0.92)	2.7 (0.73–10.0, 0.14)
Seizure	2	14.3	1.06 (0.26–4.27, 0.93)	0.93 (0.22–3.88, 0.92)
Decreased level of consciousness	0	0	na	
Papilledema	5	35.7	1.16 (0.51–2.62, 0.71)	2.05 (0.84–4.95, 0.11)
Other	0	0	na	
Incidental finding	0	0	na	
Thrombus location				
Isolated sinus	3	21.4	na	
Jugular vein	1	0.7	na	
Extensive thrombosis	7	50	0.76 (0.4–1.4, 0.4)	1.05 (0.58–1.88, 0.87)
Deep or cortical veins	3	21.4	na	
Treatment				
Warfarin or LMWH	13	92.8		
None	1	0.7		
Adverse clinical outcome				
Intracranial hemorrhage	5	35.8	2.5 (1.17–5.36, **0.02**)	1.88 (0.79–4.47, 0.16)
Surgical intervention	2	14.3	1.6 (0.43–6.35, 0.46)	4.5 (0.69–29.27, 0.12)
Epileptic disorder	2	14.3	1.31 (0.34–4.99, 0.68)	1.13 (0.27–4.73, 0.16)
Venous infract	0	0	na	
Recurrent thrombosis	1	7.6	na	
Recurrent hospitalization	4	28.6	1.7 (0.71–4.05, 0.23)	1.8 (0.66–4.91, 0.25)
Imaging outcome (6–12 months follow-up)				
No recanalization	1	0.7	na	
Partial recanalization	5	35.8	1.8 (0.8–3.7, 0.12)	1.73 (0.73–4.05, 0.21)
Missing data	3	21.4		

# *1—Factor V Leiden heterozygous, 1—Active malignancy*.

* *The overrepresentation of female gender did not pose a significant risk factor for thrombosis*.

*ns, non significant; na, not applicable due to small sample size; CTA, computed tomography angiography; MRA, magnetic resonance angiography; LMWH, low-molecular-weight heparin; DOAC's, direct oral anticoagulants; RR, relative risk, CI, confidence interval. Bold values refers to statistically significant values*.

**Table 4:** Subgroup analysis of APLA-positive patients.

**Demographics and clinical data APLA positive patients (*n* = 23)**	**N**	**%**	**RR (CI, *p* value)**
Mean age = 41 ± 15 y			
Median age = 39 y			ns
Female^**^	18	78.3	2.14 (1.66–2.78, **<0.001**)
Smoking	3	13.0	0.95 (0.31–2.84, 0.92)
Underlying chronic conditions	4	17.4	1.79 (0.68–4.72, 0.24)
Oral contraceptives	4	17.4	1.05 (0.42–2.7, 0.9)
Symptoms and signs		0.0	
Headache	14	60.9	2.07 (1.35–3.16, **0.0007**)
Visual disturbances	1	4.3	na
Focal signs	4	17.4	1.8 (0.62–5.18, 0.28)
Seizure	0	0.0	1.81 (0.47–7.02, 0.86)
Decreased level of consciousness	1	4.3	na
Papilledema	7	30.4	1.9 (0.93–4.17, 0.07)
Other	1	4.3	na
Incidental finding	1	4.3	na
Thrombus location			
Isolated sinus	1	4.3	na
Jugular vein	2	8.7	na
Extensive thrombosis	15	65.2	1.3 (0.75–2.2, 0.38)
Deep or Cortical veins	1	4.3	na
Treatment			
Warfarin or LMWH	23	100.0	
None			
Adverse clinical outcome			
Intracranial hemorrhage	2	8.7	1.47 (0.64–3.37, 0.36)
Surgical intervention	4	17.4	2.12 (0.79–5.71, 0.13)
Epileptic disorder	2	8.7	0.77 (0.19–3.04, 0.71)
Venous infract	1	4.3	na
Recurrent thrombosis	0	0	na
Recurrent hospitalization	6	26.1	1.55 (0.74–3.26, 0.24)
Imaging outcome (6–12 months follow-up)			
No recanalization	3	13.0	na
Partial recanalization	7	30.4	1.5 (0.78–2.91, 0.23)
Missing data	2	8.7	

** *The overrepresentation of female gender did not pose a significant risk factor for thrombosis*.

*ns, non-significant; na, not applicable due to small sample size; CTA, computed tomography angiography; MRA, magnetic resonance angiography; LMWH, low-molecular-weight heparin; DOAC's, direct oral anticoagulants; RR, relative risk; CI, confidence interval. Bold values refers to statistically significant values*.

In terms of JAK-2 mutation, the proportion of patients screened for the mutation was 32.7% (77 out of 236 patients). The indications for screening were blood count abnormalities, followed by idiopathic and recurrent or extensive thrombosis. Among the screened patients, 14 were JAK-2 positive (18.2% positivity rate), with only one patient presenting with a prediagnosed ET; 12 of the 14 patients were females (85.7%). A total of 5 (35.7%) screened patients had normal blood counts on presentation, 3 (21.4%) were smokers, and 2 (14.3%, representing 16.6% of female patients) used oral contraceptives. Only 1 patient was presented with underlying malignancy, and 1 patient was tested positive for the alternative hypercoagulable state (FVL). In the newly diagnosed JAK-2 positive cases *with* abnormal blood counts, all had an isolated thrombocytosis, *without* erythrocyte count abnormality.

A focus on the JAK-2 and APLA positive subgroups did not reveal a significant trend for clinical or surgical complications in comparison to the general cohort (though the analysis was confounded by a small number of final cases). Papilledema, intraparenchymal hemorrhage, headaches, focal signs, or seizure presentation rates were not overall higher in this group in comparison to the general cohort, with only a trend toward worse radiological or long-term clinical outcomes. A statistically significant difference was found only in the headaches and ICH presentation in the JAK-2 group and headache presentation in the APLA group (Tables 3, 4). However, in our cohort, the 3 most devastating cases were associated with the JAK-2 positivity. These cases required either urgent thrombectomy or resulted in long-term epilepsy or long-standing papilledema that required recurrent intervention. One of these cases was presented with a normal blood count. It should be noted that in the majority of the JAK-2 positive cases (12 of 14, 86%), the mutation was the only diagnosed etiology of thrombosis, rendering it as a sole and independent risk factor for thrombosis. In a demographic comparison, the JAK-2 group did differ from the overall cohort in terms of female gender overrepresentation, with a limitation of a smaller, underpowered sample size.

Other cases that resulted in acute interventions or neurological long-term sequelae were diagnosed with antiphospholipid syndrome (new or previously known, 4 cases), local cranial or CNS infection (4 cases), prothrombin mutation (2 cases), and Behcet's disease (1 case).

## Discussion

Sinus venous thrombosis is a rare subtype of cerebral thrombosis, with a potential for devastating outcomes. In our cohort, we show that in concordance with the previously published data, in ~39% of cases the cause of the thrombosis is idiopathic (18), while the most common identifiable causes of cerebral venous thrombosis are acquired or genetic thrombophilias. Thus, APLA, FV Leiden, prothrombin mutation, protein S or C abnormalities, and malignancy led to the etiological chart. Additionally, the overall demographic characteristics of our cohort agree with the previously accepted knowledge on CSVT (19, 20). There was a higher prevalence of CSVT in the female patients, in whom ~33% were on the concurrent oral contraceptive treatment. The most common presenting symptom in our population was a headache, followed by papilledema and visual disturbances. In keeping with current guidelines (21), the majority of the patients were treated successfully with either warfarin or low-molecular-weight heparin. Finally, the most common thrombosis locus involved a superficial venous system, with a minority of the cases presenting with deep/cortical or cervical venous thrombosis.

In our cohort, only a small fraction of patients tested positive for the JAK-2 somatic mutation; however, the overall positivity rate was high since not all patients were screened. The JAK-2 screening rationale was based on the accepted practice to include patients that present either with abnormal blood counts or with recurrent and/or idiopathic thrombosis (22, 23). However, as we show in this cohort, the JAK-2 positive cases included also first-ever thrombosis cases presenting with normal blood counts.

Bone marrow abnormalities associated with the JAK-2 somatic mutation represent an important subset of hematological disorders, with various systemic manifestations, including CNS. Early diagnosis of these conditions is important, both with respect to malignant transformation monitoring and early cytoreductive therapy in the context of an unprovoked thrombotic event (24–26). Vigilant blood count monitoring is warranted following the JAK-2 diagnosis, with potential subsequent bone marrow sampling, due to suspected malignant transformation or development of myelofibrosis (24, 25). With respect to tailored antithrombotics combined with hematologic treatment in the context of systemic thrombosis, the JAK-2 mutation discovery might warrant a long-standing anticoagulant or at least antiplatelet therapy, specifically in the older patients (65 years old and above), in patients with non-transient concurrent risk factors or patients with an active myeloproliferative disorder (26).

Additionally, we showed that the JAK-2-mutation-associated thrombosis was associated with a poorer clinical course in 3 otherwise healthy patients (a non-significant trend, probably due to overall low incidence). In selected cases, the JAK-2 mutation was not associated with blood count abnormalities, with the mutation being the sole independent risk factor for thrombosis (~80% of positive cases). A possible explanation of this phenomenon can be found in the recently published studies that postulate that downstream molecular mechanisms are responsible for hypercoagulability in mutation carriers, meaning that elevated blood viscosity due to increased hematocrit or thrombocytosis is not the sole contributor to thromboembolic events (11, 23, 24).

These findings, together with the data presented here, suggest that the JAK-2 mutation might be underdiagnosed in the cases of idiopathic cerebral venous thrombosis, regardless of blood count status. In our study, the positivity rate for JAK-2 mutation reached 13%, which is less than previously reported in other cases of systemic venous thrombosis (23) but higher than expected, especially in the cases of first-ever thrombosis with normal blood counts. This notion is of extreme importance in the workup of cerebral venous thrombosis in terms of widening therapeutic options to include tailored treatment beyond just anticoagulation—hence improving clinical outcomes—as well as in the potential prevention of hematologic malignancy. The major limitation of this study was limited screening for the JAK-2 mutation, making the study prone to selection bias. The conclusion of a more severe course trend is of course biased as well since that was one of the criteria for JAK-2 screening. Another limitation of the retrospective nature of the study is that we relied only on the accepted departmental protocols for acute sinus vein thrombosis imaging. These protocols currently rely mostly on CT venography, sometimes combined with a simple MR gadolinium enhanced venography, while catheter angiography is reserved for the cases that require an urgent venous thrombectomy. Of course, a future prospective study would include advanced vessel wall imaging. It should be noted as well that this study was performed in the mixed Mediterranean and European Jewish and Arab populations, maybe limiting the prevalence of the JAK-2 mutation in this group. However, no ethnicity-specific correlation is known or has been published since the discovery of the mutation in 2005 (6, 27, 28). Interestingly, in our JAK-2-positive CSVT patients, there was an overwhelming overrepresentation of female patients (86%), with an underrepresentation of oral contraceptive use in this group (15% compared to 33% in the total female cohort), further strengthening the notion of JAK-2 somatic mutation being an independent risk factor for thrombosis (29, 30). Additionally, it is important to note that these findings are not essentially new since previous reports from a decade ago and earlier had indicated an association between JAK-2 mutation and cerebral venous thrombosis, also without laboratory evidence for the myeloproliferative disorder (31–34).

**Figure 1:**
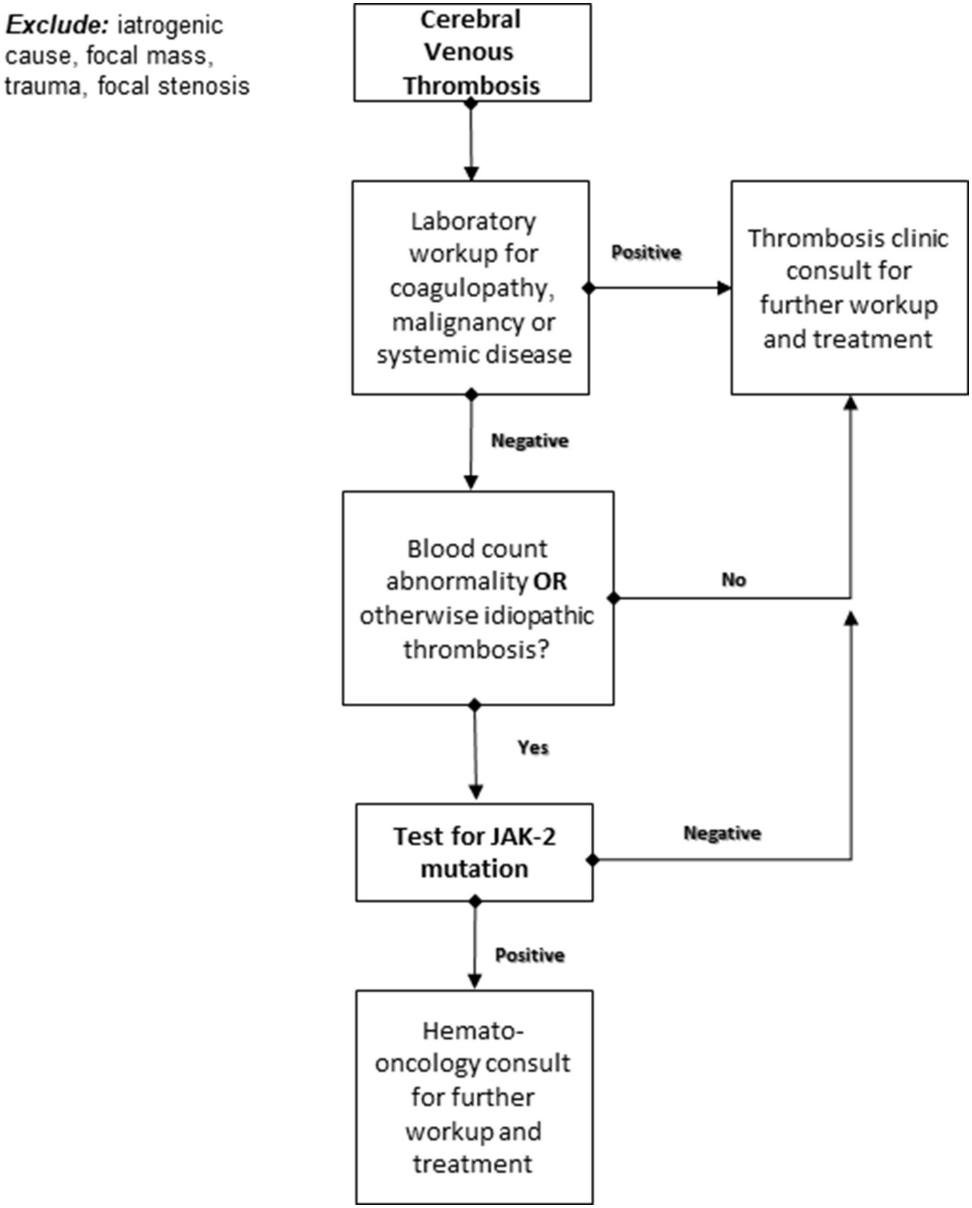
Proposed decision-making algorithm for JAK-2 somatic mutation testing following an acute cerebral venous thrombosis.

The results of the current cohort indicate the need to further inquire as to the possible connection between the mutation and worse thrombosis outcomes; they also indicate a higher than previously estimated JAK-2 positivity rate in the cerebral venous thrombosis. Based on these findings, we propose a specific screening decision-making flow chart that would allow better patient selection for JAK-2-mutation screening (Figure 1).

In conclusion, the JAK-2 mutation is still underdiagnosed in the cases of idiopathic thrombosis. Larger prospective cohorts are needed that would include systematic screening for JAK-2 mutations in all the cases of idiopathic cerebral venous thrombosis. This will enable a revision of current workup and treatment guidelines.

## Data Availability Statement

The raw data supporting the conclusions of this article will be made available by the authors, without undue reservation.

## Ethics Statement

The retrospective evaluation of patient files was approved by the Ethical Committee of the CSMC and RMC (Helsinki Committee Approval Numbers 6067-19, 0452-18, respectively). Written informed consent for participation was not required for this study in accordance with the national legislation and the institutional requirements.

## Author Contributions

DO and ZI-H contributed equally to the design of the study and final manuscript drafting. ZI-H, SP, and RM contributed to data collection and analysis. GY and MA contributed to the imaging and clinical revision of the data. DG-M provided scientific supervision to the project and manuscript preparation. All authors contributed to the article and approved the submitted version.

## Conflict of Interest

The authors declare that the research was conducted in the absence of any commercial or financial relationships that could be construed as a potential conflict of interest.

## Publisher's Note

All claims expressed in this article are solely those of the authors and do not necessarily represent those of their affiliated organizations, or those of the publisher, the editors and the reviewers. Any product that may be evaluated in this article, or claim that may be made by its manufacturer, is not guaranteed or endorsed by the publisher.
